# Bilateral Mandibular Premolar Dens Invaginations: A Case Report

**DOI:** 10.1155/2012/474013

**Published:** 2012-10-09

**Authors:** Richard Holliday, Emma Beecroft

**Affiliations:** ^1^Restorative Dentistry Department, Newcastle Dental Hospital, Newcastle upon Tyne NE2 4AZ, UK; ^2^General Dental Practitioner and Associate Clinical Lecturer, Newcastle Dental Hospital, Newcastle upon Tyne NE2 4AZ, UK

## Abstract

Dens invaginations are a rare developmental defect most commonly affecting maxillary lateral incisors, with very few reported cases in mandibular teeth. We describe a rare case of bilateral first mandibular premolar dens invaginations type I, where apparently health teeth presented with periapical pathology.

## 1. Case Report

**Figure 1:**
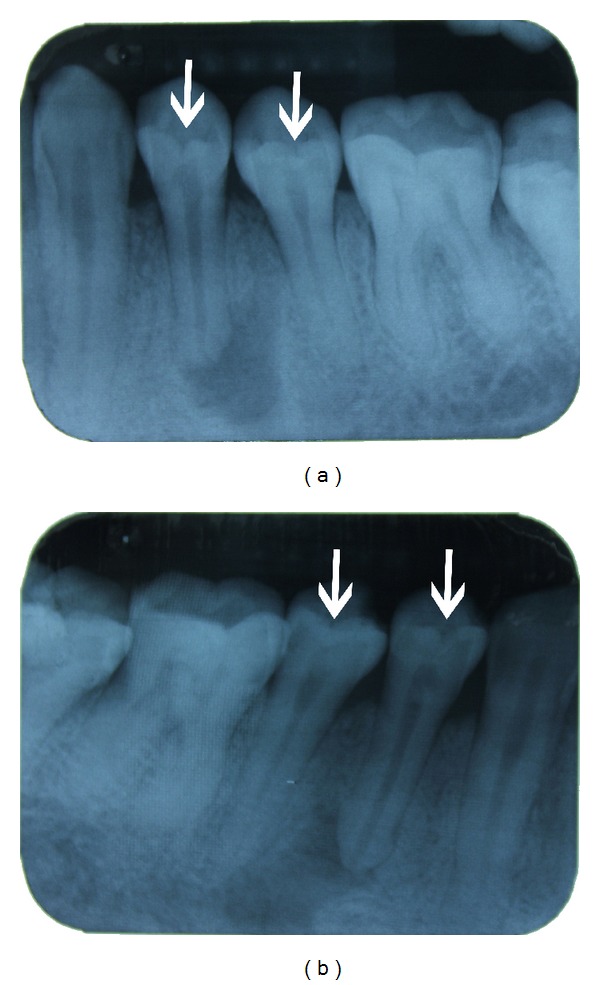
Long-cone periapical radiographs showing the left 1(a) and right 1(b) mandibular premolars. Well-demarcated periapical radiolucencies can be seen associated with the first premolar teeth bilaterally. The white arrows indicate radiolucent “invaginations” on the occlusal surfaces of the teeth.

A 25-year-old medically fit and well male patient presented twice within a two-week period at a dental emergency clinic. First presentation was due to a 7-day history of constant aching pain affecting the left side of his face, and the patient could isolate the pain to the left mandible. Extraoral and intraoral soft tissue examination was unremarkable. Periodontally there were no pathological pockets, and oral hygiene was good. Dentally the patient had an unrestored adult dentition with absent third molars and no detectable caries. The lower left first premolar tooth was markedly tender to percussion, and a long-cone periapical radiograph (LCPA) revealed a significant periapical radiolucency (Figure 1(a)). A diagnosis of acute apical periodontitis was confirmed although the cause of loss of vitality was unexplained, without apparent coronal or periodontal pathology being identifiable. The patient was offered extraction or pulp extirpation, and the patient opted to save the tooth. The tooth was successfully accessed and dressed with ledermix, cotton wool, and glass ionomer cement (GIC). The patient was discharged to find a general dental practitioner (GDP) to complete root canal treatment. 

Two weeks later, the patient reattended the same dental emergency clinic complaining of constant aching pain affecting the lower right mandible. Once again, no decay or periodontal issues were found clinically, but LCPA examination of the lower right quadrant showed periapical pathology associated with the mandibular right first premolar tooth (Figure 1(b)). A diagnosis of acute apical periodontitis was confirmed, and the patient again had pulp extirpation completed.

## 2. Differential Diagnosis

With two teeth loosing vitality without any obvious coronal or periodontal pathology, our differential diagnosis included trauma or developmental defects. There was no history of trauma, and indeed this site would be an unusual location for a traumatised tooth, usually affecting more anterior teeth. Developmental defects were suspected, and on examination of the radiographs, radiolucent voids were seen on the occlusal surfaces of the premolar teeth. Our diagnosis was of dens invaginations (DIs) of the first mandibular premolars (+/− second premolars) although this could only be definitely confirmed by extracting and sectioning the teeth or exposing a computerised tomography (CT) scan, both of which were felt not to be justified. The diagnosis was explained to the patient and they were encouraged to register with a GDP for definitive RCT of 34 and 44. We also suggested further investigations of the vitality of the 35 and 45 and placement of fissure sealants in a bid to seal and protect these teeth.

## 3. Discussion

DI is a dental developmental abnormality arising during early odontogenesis and before calcification. Invagination of a portion of the enamel organ into the dental papilla forms infoldings lined by enamel into the crown of the tooth [1, 2].

DI's are most commonly classified into three groups depending on the depth of invagination. In type I (79%), the invagination is limited to the crown, not extending past the amelocemental junction (ACJ). In type II (15%), the invagination extends past the ACJ into the root but does not communicate with the periodontal ligament. In type III (5%), the invagination extends through the root and either connects with the periodontal ligament laterally (type IIIA) or apically through the apical foramen (type IIIB) [1, 2]. 

Embryology, trauma, and infection have been suggested as potential causes of DI, but the aetiology remains disputed [1, 2]. What is undisputed however is that the most commonly affected teeth are maxillary teeth with the permanent lateral incisors having the highest prevalence of up to 10% [1, 2]. Invaginations can be bilateral with rates of up to 43% discussed [1, 2]. There are a small number of papers [3] documenting DI presentation in mandibular teeth; however, studies show very low or in fact zero prevalence rates [1].

Clinically DI is difficult to diagnose as coronal anatomy can appear normal, as in this reported case. Signs suggestive of DI include deep foramen caecum, exaggerated cingulum pits, penetrating fissures, grooved palatal enamel, talon cusps, and incisal notching [1, 4]. Gross crown malformations such as peg- or barrel-shaped teeth have an increased association with DI [4]. 

Radiographic features can be easier to distinguish. The following appearances have been described: alterations in enamel morphology, radiolucent pocket, tear-shaped loop, undilated fissure, pseudocanal, blunting of pulp horns, and gross alteration of internal anatomy of the crown or root [2, 4].

If DI is suspected, early intervention is advocated to prevent pulpal necrosis. Fissure sealant or flowable composite application to suspect areas of newly erupted teeth is essential [2, 4]. If this is not completed, early loss of vitality is common leading to incomplete root formation. Full mouth radiographic examination should be considered after a diagnosis of DI due to the high prevalence of bilateral occurrence [1, 2].

If, like in this case, DI is only diagnosed following pulpal necrosis, endodontic therapy to save the tooth is required. This may require specialist referral as endodontic treatment of DI-affected teeth is often complex due to altered internal anatomy of affected teeth [4]. MTA apical barriers may need to be used in cases with open apices subsequent to immature root formation [1, 4].

## 4. Conclusion

This paper highlights a rare presentation of bilateral mandibular premolar DI and shows that although DI affected teeth are anatomically abnormal, clinically this can be difficult to detect. Despite this difficulty, all general dental practitioners should be aware of the possible clinical and radiographic signs, should carefully evaluate all suspect teeth, and where appropriate treat prophylactically with a view to preventing loss of pulp vitality.
